# Whey protein supplementation and exercise in older adults: a narrative review of potential effects on muscle–brain axis, physical function, and related outcomes

**DOI:** 10.3389/fnut.2026.1772577

**Published:** 2026-07-10

**Authors:** ChuXin SiMa, ShiJie Zhang, HuiZheng Ma, Rongjie Wu, Yong Liao

**Affiliations:** 1 Department of Sports Medicine, Dongshin University, Naju, Republic of Korea; 2 School of Public Health, Lanzhou University, Lanzhou, Gansu, China; 3 Graduate School of General, Dongshin University, Naju, Republic of Korea

**Keywords:** aging, cognitive function, exercise, physical performance, whey protein

## Abstract

Aging is associated with progressive declines in cognitive function, physical performance, and musculoskeletal integrity, increasing the risk of frailty and injury. Recent evidence highlights the importance of the muscle–brain–metabolism axis in mediating these age-related changes. Interventions targeting this axis, including whey protein supplementation combined with structured exercise, have attracted growing attention. This narrative review summarizes current evidence regarding the combined effects of whey protein and exercise on physical performance, muscle health, and selected biomarkers in older adults, while also discussing emerging findings related to cognitive function and injury risk. Mechanistically, these interventions may support muscle protein synthesis, modulate inflammatory pathways, improve metabolic regulation, and influence neurotrophic signaling. However, current evidence regarding cognitive outcomes and injury prevention remains limited and, in some cases, inconsistent, with many studies reporting these outcomes as secondary endpoints. Therefore, while combined interventions show potential for improving aspects of healthy aging, further well-designed studies are required to establish their effectiveness in these domains. Overall, targeting the muscle–brain axis through nutritional and exercise-based strategies represents a promising but still evolving approach for supporting healthy aging and functional capacity in older adults.

## 1 Introduction

The process of aging is characterized by a gradual reduction in skeletal muscle mass, strength, neuromuscular coordination, metabolic adaptability, and cognitive capabilities, which collectively contribute to increased frailty, disabilities, and a diminished capacity for independence. A significant biological characteristic that underpins these transformations is anabolic resistance, which is characterized by the reduced capacity of aging muscle to adequately respond to anabolic stimuli, including dietary protein consumption and physical exercise (1, 2). Within this framework, the integration of nutritional strategies alongside physical exercise has emerged as a fundamental methodology to mitigate functional deterioration. Specifically, whey protein supplementation has garnered significant scholarly attention owing to its rapid absorption characteristics, elevated leucine concentration, and its profound ability to stimulate muscle protein synthesis (MPS) through the activation of the mammalian target of rapamycin complex 1 (mTORC1) signaling pathway (3, 4). Conversely, resistance training (RT) is recognized as the most efficacious non-pharmacological intervention for enhancing muscle mass, strength, and neuromuscular function among older adults, concurrently improving insulin sensitivity and overall metabolic health (5, 6).

Most extant systematic reviews and meta-analyses predominantly concentrate on sarcopenia-associated endpoints such as lean body mass (LBM), handgrip strength, or physical performance, frequently amalgamating heterogeneous protein sources and intervention protocols (7, 8). This methodology obfuscates the specific effects attributable to whey protein and constrains mechanistic interpretations, given that whey protein exhibits considerable divergence from alternative protein sources in terms of amino acid kinetics, bioactive peptide composition, and endocrine responses. Furthermore, previous reviews infrequently synthesize cognitive outcomes, injury prevention, or molecular biomarkers within a cohesive analytical framework, opting instead to regard muscle, cognitive function, and metabolic health as predominantly separate entities. Consequently, the potential for synergistic, system-wide interactions resulting from whey protein supplementation and exercise regimens incompletely understood rather than definitively established. When evaluating cognitive outcomes in trials involving whey protein or physical exercise, these assessments are often relegated to secondary endpoints, employing a diverse array of neuropsychological assessments alongside relatively brief intervention periods. Recent randomized controlled trials (TCTs) exemplify this limitation, documenting modest enhancements in specific executive functions attributable to whey protein supplementation, yet revealing no consistent additive or synergistic effects when coupled with RT (9). These findings should be interpreted cautiously, as they may reflect both true biological limitations and methodological constraints.

The nascent notion of the muscle–brain–metabolism axis offers a comprehensive framework through which these seemingly unrelated findings may be understood. Skeletal muscle has now been acknowledged as an endocrine organ that interacts with the brain and various metabolic tissues via myokines, exerkines, metabolites, and inflammatory mediators (3, 5). Factors induced by exercise, such as irisin, brain-derived neurotrophic factor (BDNF), and modifications in amino acid and glucose metabolism, provide credible mechanistic connections between peripheral muscular activity and central nervous system functionality. Whey protein, through its modulation of amino acid availability, oxidative stress, and inflammatory response, may further impact this inter-organ communication network. Nevertheless, human trials infrequently evaluate these molecular mediators in conjunction with functional outcomes, resulting in significant deficiencies in causal inference and translational relevance. Another significant constraint of the extant reviews is the notable scarcity of outcomes related to injury prevention and recovery. Although improvements in muscle function and metabolic resilience may theoretically reduce risk factors associated with injury (e.g., falls or impaired recovery), direct evidence supporting a clear role of whey protein supplementation in injury prevention remains limited. Nevertheless, metrics such as injury incidence, fall rates, and biomarkers indicative of musculoskeletal repair are infrequently recognized as primary outcomes in studies pertaining to whey protein and exercise, and are predominantly neglected in previous syntheses (10). This oversight considerably diminishes the clinical applicability of the current evidence, especially for elderly populations in which injury prevention constitutes a fundamental therapeutic objective.

The existing corpus of scholarly literature is marked by robust mechanistic plausibility, moderate yet heterogeneous evidence supporting enhancements in physical performance, and preliminary, inconsistent outcomes regarding cognitive functions and injury-related metrics. Prior comprehensive reviews have not systematically synthesized the effects of whey protein supplementation and exercise on cognitive abilities, physical performance, injury prevention, and biomarker modulation within the theoretical construct of the muscle–brain–metabolism axis. Consequently, the present review is imperative for addressing these deficiencies, rigorously evaluating discrepancies, and differentiating evidence-based conclusions from speculative hypotheses, while specifically considering whey protein as a distinct and biologically pertinent nutritional intervention. Several salient messages arise from the extant evidence and experiential knowledge within the discipline. First, the amalgamation of RT with sufficient whey protein consumption appears to be an effective strategy to augment muscular strength and specific functional outcomes in the geriatric population. Second, while the cognitive advantages are theoretically tenable, current human evidence remains insufficient to support definitive conclusions regarding synergistic cognitive enhancement beyond the well-documented effects attributable to exercise in isolation. Third, the mechanistic pathways that connect muscular adaptations to cerebral and metabolic health remain insufficiently characterized in human studies, thereby necessitating a prudent interpretation of the findings. Finally, the variability among individuals concerning anabolic resistance, metabolic health, and foundational nutritional status highlights the imperative for tailored intervention strategies as opposed to generalized recommendations. Future investigations ought to prioritize robustly powered TCTs featuring co-primary physical and cognitive endpoints, standardized formulations of whey protein, alongside comprehensive longitudinal assessments of molecular mediators that encompass muscular, metabolic, and neurological domains. Special consideration should be directed towards high-risk demographics, including frail, insulin-resistant, or cognitively vulnerable elderly individuals, in addition to clinically significant outcomes such as the incidence of falls, rates of injury, and the preservation of functional independence. The integration of biomarker-driven mediation analyses with functional outcomes is imperative for the translation of the muscle–brain–metabolism axis from a theoretical construct into a clinically applicable framework conducive to promoting healthy aging.

## 2 Literature search strategy and scope of the narrative review

### 2.1 Characteristics of included studies

This article is designed as a narrative review aiming to provide an integrative overview of current evidence regarding the synergistic effects of whey protein supplementation and physical exercise in older adults, with a particular focus on mechanistic pathways related to the muscle–brain axis. Relevant studies were identified through searches of electronic databases, including PubMed, Scopus, and Web of Science. The literature search was conducted using combinations of keywords such as “whey protein,” “exercise,” “aging,” “sarcopenia,” “muscle protein synthesis,” “cognitive function,” and “muscle–brain axis.” In addition, the reference lists of selected articles were manually screened to identify further relevant studies. This approach was intended to enhance the comprehensiveness of the evidence base and minimize the risk of selection bias. Studies were selected based on their relevance to the topic according to a structured narrative selection framework designed to ensure transparency and reduce selection bias. The studies included in this review were identified using predefined eligibility criteria to improve methodological clarity and consistency. Specifically, inclusion criteria comprised human studies involving older adults (generally aged ≥60 years, or as defined by the original study populations, typically ranging between 60 and 85 years), as well as intervention studies evaluating resistance training, aerobic exercise, or multicomponent exercise combined with whey protein or related protein-based supplementation, including leucine-enriched or multi-ingredient formulations. Eligible study designs included randomized controlled trials, quasi-experimental studies, and controlled intervention studies. Furthermore, studies were required to report at least one relevant outcome related to muscle function (such as strength, muscle mass, or physical performance), metabolic biomarkers (including inflammatory markers, lipid profile, or insulin resistance indices), or molecular and cellular adaptations (such as muscle protein synthesis, mTOR signaling, or protein turnover). Both acute mechanistic studies and long-term interventions ranging from short-term experimental protocols to extended training programs of up to 12–24 months were considered.

Exclusion criteria included animal or *in vitro* studies, non-English publications, review articles, editorials, or opinion papers lacking original data, as well as studies without a clear exercise or protein-related intervention component. The studies discussed in this narrative review predominantly encompass randomized controlled trials, quasi-experimental frameworks, and longitudinal intervention studies evaluating the independent or synergistic effects of whey protein supplementation and physical exercise in geriatric populations. Randomized controlled trials with parallel or crossover designs are emphasized where available due to their higher internal validity and ability to support causal inference. Across studies, intervention duration ranged from acute experimental protocols assessing post-exercise metabolic and molecular responses to extended training programs lasting 8 to 52 weeks, and in some cases up to 12–24 months, thereby allowing evaluation of functional, metabolic, and molecular adaptations.

Key methodological features across the included literature involved supervised exercise training, adherence monitoring, regulation of habitual dietary protein intake, and careful timing of protein ingestion relative to exercise sessions. Outcome assessments were multidimensional, including measures of physical performance, body composition, metabolic biomarkers, and molecular signaling pathways such as muscle protein synthesis and inflammatory regulation, all of which are relevant for understanding interactions within the muscle–brain–metabolism axis. However, given the narrative nature of this synthesis, heterogeneity in study design, sample size, intervention protocols, and protein dosage should be considered when interpreting the findings. A structured and transparent selection approach was therefore implemented to minimize subjective bias while preserving the integrative scope of a narrative review.

### 2.2 Population profiles (healthy, frail, sarcopenic, clinical cohorts)

The reviewed literature included heterogeneous aging populations with varying functional, metabolic, and cognitive profiles. Healthy older adults were frequently studied to evaluate preventive strategies aimed at maintaining cognitive performance, muscle strength, and metabolic health during normal aging. Frail and sarcopenic populations represented a key focus due to their heightened vulnerability to anabolic resistance, functional decline, and injury risk. In these cohorts, combined exercise and whey protein interventions were examined for their potential to improve muscle mass, neuromuscular coordination, and functional independence. Diagnostic criteria for frailty and sarcopenia varied across studies, commonly incorporating muscle mass, strength, and physical performance indices. Additionally, several studies investigated clinical cohorts, including older adults with mild cognitive impairment, metabolic disorders, or chronic inflammatory conditions. These populations provided insight into how disease-related metabolic dysregulation and neuroinflammation may influence responsiveness to combined nutritional and exercise-based interventions. However, evidence across these subpopulations remains heterogeneous, and findings should be interpreted with consideration of study design differences and population-specific characteristics.

### 2.3 Types of exercise and whey protein formulations

Exercise interventions displayed considerable heterogeneity in terms of modality, intensity, frequency, and structural organization among the reviewed studies. RT emerged as the preeminent modality utilized, due to its critical function in promoting MPS and augmenting muscular strength through the activation of anabolic signaling pathways, specifically the PI3K/Akt/mTOR cascade (8, 11). Aerobic exercise modalities, encompassing both continuous and interval-based protocols, were frequently integrated with RT to potentially enhance cardiovascular fitness, cerebral perfusion, mitochondrial function, and metabolic regulation (12, 13). Furthermore, multicomponent exercise programs that incorporate balance, flexibility, and functional training were particularly pertinent for frail older adults due to their possible contributions to mobility preservation, functional autonomy, and reduction of fall risk, although direct evidence concerning injury prevention remains scarce in several investigations (14). Whey protein supplementation protocols exhibited substantial variability concerning formulation types, leucine concentrations, dosages, digestion kinetics, and the timing of administration. The majority of investigations administered whey protein doses ranging from 15 to 40 grams per serving, which corresponds with the suggested anabolic threshold necessary to optimize postprandial MPS in the elderly population (15, 16). Various formulations including whey protein concentrate (WPC), whey protein isolate (WPI), and whey protein hydrolysate (WPH), were employed across these studies and may exert differential influences on physiological outcomes. WPC contains bioactive compounds alongside moderate levels of lactose and fat, whereas WPI offers enhanced protein purity with diminished concentrations of carbohydrates and lipids. Conversely, WPH undergoes enzymatic hydrolysis, leading to accelerated amino acid absorption and potentially greater stimulation of acute MPS responses (17). Numerous investigations have utilized leucine-enriched whey formulations to mitigate age-related anabolic resistance through the enhanced activation of mTOR-dependent signaling pathways (18, 19). The variability present in amino acid composition, digestibility, absorption kinetics, and leucine bioavailability may, thus, partially elucidate the discrepancies observed in muscle-related and neurocognitive outcomes across different studies. Moreover, the timing of whey protein consumption constituted an additional critical methodological variable. Certain studies administered supplementation immediately prior to or following exercise sessions to optimize anabolic sensitivity, while others allocated protein intake uniformly throughout the day to enhance net protein balance and metabolic regulation (20). Evidence indicates that protein timing may not only affect skeletal muscle adaptation but also influence neurotrophic signaling, inflammation, and the communication axis between muscle and brain in aging populations (21). Consequently, variations in exercise modalities and whey protein formulations must be taken into account when interpreting the heterogeneity of outcomes documented across the studies included.

## 3 Effects of whey protein and exercise on skeletal muscle health

**Figure 1:**
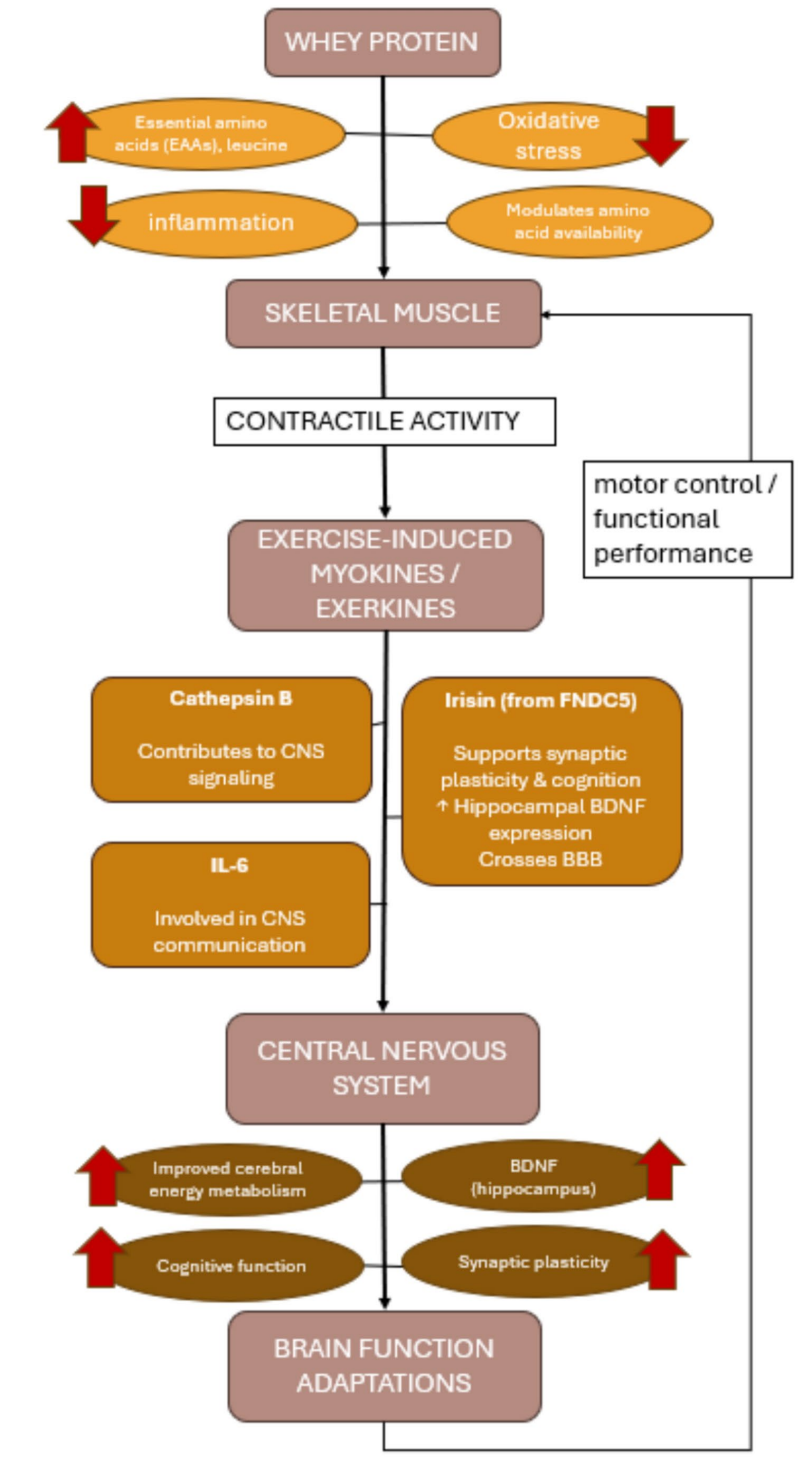
Integrated muscle–brain–metabolism axis linking exercise and whey protein supplementation. This schematic illustrates how skeletal muscle functions as an endocrine organ connecting peripheral muscle activity with the central nervous system and metabolic tissues. Whey protein provides essential amino acids—particularly leucine—modulating amino acid availability, oxidative stress, inflammation, and muscle-specific microRNA (myomiR) regulation. Exercise-induced muscle contraction stimulates the release of myokines and exerkines, including irisin (derived from FNDC5), cathepsin B, and IL-6, which contribute to CNS signaling. Irisin may cross the blood–brain barrier and is associated with increased hippocampal BDNF expression, supporting synaptic plasticity and cognitive function. Muscle-derived myomiRs regulate myogenesis, satellite cell activation, protein turnover, and target key signaling cascades such as mTOR, FoxO, and NF-κB, thereby integrating the effects of exercise and whey protein on anabolic pathways. Enhanced PI3K/Akt/mTORC1 signaling promotes muscle protein synthesis and muscle remodeling. Exercise and whey protein jointly influence systemic metabolic pathways, including amino acid and glucose metabolism, while exercise-induced improvements in insulin sensitivity and mitochondrial function contribute to more efficient cerebral energy metabolism. Together, these mechanisms illustrate a bidirectional muscle–brain–metabolism axis that supports improved muscle function, cognitive performance, and overall metabolic health.

Anabolic resistance associated with aging, obesity, sarcopenia, and metabolic dysfunction is a major contributor to the progressive decline in muscle mass, muscle strength, muscle quality, and physical function. Collectively, the available evidence indicates that RE is the principal stimulus driving improvements in skeletal muscle health, whereas the additional benefits of whey protein supplementation appear to be influenced by participant characteristics, baseline nutritional status, protein quality, exercise intensity, and metabolic status (Figure 1).

A substantial body of evidence supports beneficial effects of combining whey protein supplementation with exercise interventions, particularly in older adults exhibiting anabolic resistance or elevated cardiometabolic risk. Studies conducted in sarcopenic, obese, frail, metabolically compromised, or recently hospitalized populations demonstrated improvements in appendicular lean mass, muscle quality, handgrip strength, lower-limb strength, mobility, and functional performance when whey protein was incorporated into exercise programs (22–29) (Table 1). Furthermore, whey protein supplementation was associated with favorable changes in body composition, including reductions in fat mass and improvements in metabolic profiles, whereas resistance exercise contributed to increases in fat-free mass and resting energy expenditure. Evidence further suggests that the combination of exercise and whey protein may promote greater reductions in adiposity than exercise alone (30). In addition, beneficial effects on selected cardiometabolic risk markers, including fasting insulin, insulin resistance, and LDL cholesterol, have also been reported (25). These findings suggest that nutritional support may enhance exercise responsiveness in populations characterized by impaired anabolic sensitivity.

**Table 1:** Overview of conducted studies on protein supplementation, resistance training, and muscle function in older adults.

Population/Sample size	Intervention	Duration	Outcomes	Key statistical results	Ref
Free-living older men (*n* = 32); subgroup with overweight/obesity and ≥1 metabolic syndrome (MetS) risk factor	Resistance exercise + daily PA + multi-ingredient supplementation (PLA vs. M5: whey/casein + creatine + vit D + fish oil)	3 months	M5: Lean mass ↑, strength ↑, performance ↑, bone markers ↑; PLA: Lean mass ↓, strength ↔/↓, performance ↓; Obesity/MetS: anabolic response ↓	Obesity/MetS negatively correlated with response (r = −0.36 to −0.68, *p* < 0.05); M5 > PLA: ASM + 2% vs. − 0.8%, strength +17% vs. − 1.4%, performance −10.5% vs. + 1.1% (all *p* < 0.05); P1NP ↑ in M5 (*p* = 0.036); overall response *p* = 0.013	(22)
Older adults with non–insulin-dependent type 2 diabetes (*n* = 39)	Resistance training twice weekly + whey protein supplementation (33 g on training days) vs. placebo	12 weeks	Strength ↑ (both) ↔ (WP effect), body composition ↑ (both) ↔, glycemic control ↔, urea ↑ (WP), QoL ↑ (small)	No added WP effect; urea *p* = 0.05	(31)
Older men (67 ± 4 years; *n* = 36)	Resistance exercise (2×/week) and/or whey protein (2 × 25 g/day) vs. control	12 weeks	Executive function ↑ (protein), global cognition ↑ (trend), cognition (RT) ↔, inflammation ↓ (RT), strength ↑, no synergy ↔	Exec function *P* = 0.03; TNF-α *p* = 0.02; IL-6 *p* = 0.048	(9)
Postmenopausal women (60 ± 7 years; *n* = 16)	Acute unilateral resistance exercise + fortified whey protein vs. water	Acute (0–4 h)	MPS ↑ early (0–2 h, exercise), MPS ↑ late (2–4 h, whey), cumulative MPS ↔, non-exercised leg MPS ↔, anabolic signaling ↔	Early MPS ↑ (*p* = 0.007); late MPS ↑ with whey (*p* = 0.005); no cumulative effect (*p* > 0.05)	(44)
Overweight postmenopausal women (BMI ≈ 29; *n* = 40)	Short-term energy restriction + unilateral resistance exercise + whey protein bolus (15 g vs. 35 g vs. 60 g)	Acute (6 days ER + 0–4 h testing)	MPS ↑ (35 g & 60 g > 15 g), MPS ↑ (fed & postexercise vs. basal), MPS ↔ (35 g vs. 60 g), MPS ↔ (energy restriction vs. balance at 35 g)	ERW35 and ERW60 > ERW15 (*p* = 0.013, 0.026); FED and FED-EX > BASAL (*p* < 0.001); no difference 35 g vs. 60 g (*p* = 1.000)	(43)
Healthy older adults >65 y (29 women, 37 men)	Daily whey protein or collagen or carbohydrate ± light or heavy resistance training	12 months	Basal MPS ↔, postprandial MPS ↔, muscle metabolome ↔, sex differences ↔	No significant changes in MPS or metabolome (*p* > 0.05); no sex difference (*p* = 0.75)	(32)
Older adults ≥65 y with sarcopenia (*n* = 81)	Resistance training and/or leucine-enriched whey protein supplementation	24 weeks intervention + 24 weeks de-training	ASMI ↑ and strength ↑ (RT + PRO post-intervention), between groups ↔ (post), during de-training: ASMI ↑ and strength ↑ maintained (RT + PRO > RT)	Post-intervention ↑ vs. baseline (*p* < 0.01); at 24-week de-training RT + PRO > RT (ASMI *p* < 0.05; HGS *p* < 0.01)	(23)
Community-dwelling older adults (70.6 ± 4.7 y; *n* = 61)	Vibration + resistance exercise ± high-protein diet ± omega-3 supplementation	8 weeks	Strength ↑, function (CRT) ↑ (high-protein), muscle power ↑ (men, omega-3), inflammation ↓ (men only), IGF-1 ↑ (omega-3), biomarkers ↔	Significant improvements in strength and CRT; sex-specific effects (men only for inflammation and power)	(24)
Older adults (*n* = 18)	10 weeks of whole-body resistance training + constant vs. graded whey protein intake (0.8 → 2.2 g/kg/d)	10 weeks	Lean mass ↑, strength ↑, muscle quality ↑ (time effect), protein strategy effect ↔	Time effects *p* < 0.05; no group × time differences	(33)
Healthy older men (67 ± 1 y; *n* = 33)	Resistance exercise (2×/week) ± whey protein supplementation (2 × 25 g/day) vs. control	12 weeks	FFM ↑, RMR ↑, sedentary EE ↑, SMR ↑ (RE), activity EE ↓, fat mass ↓ (PRO and RE + PRO), protein oxidation ↑, protein balance ↓, total EE ↔	RE ↑ RMR, SMR, FFM (*p* < 0.05); RE ↓ activity EE (*p* = 0.049); PRO ↓ FM and ↑ protein oxidation (*p* < 0.05); RE + PRO > RE + CON for FM ↓ (*p* = 0.04)	(30)
Older adults (60–80 y; *n* = 31)	Resistance training (2×/week) + whey protein (20 g breakfast + 20 g dinner) vs. placebo	12 weeks	Strength ↑, muscle thickness ↑, lean mass ↑, function ↑ (time effect), protein effect ↔	Significant time effects (*p* < 0.05); no between-group differences	(34)
Healthy active older men (67 ± 1 y; *n* = 36)	Resistance exercise (2×/week) ± whey protein supplementation (2 × 25 g/day)	12 weeks	Strength ↑, FFM ↑, physical function ↑, fat mass ↓, inflammation ↓ (RE), gait speed ↑ (protein), synergy ↔	RE ↑ strength and FFM (*p* < 0.001); ↓ IL-6 and TNF-α (*p* < 0.05); protein ↑ gait speed (*p* = 0.007); no interaction effects	(35)
Aging rats (12–13 months; human equivalent 60–65 y)	*W. somnifera* extract, protein cocktail, WSE + protein, whey protein, or resistance exercise	60 days	Strength ↑, muscle mass ↑, inflammation ↓ (CRP, IL-6, TNF-α), oxidative stress ↓, apoptosis ↓, antioxidant capacity ↑, glucose ↓; best effects: WSE + protein	All treatments improved markers vs. control; combination therapy most effective	(46)
Community-dwelling older adults (mean 68.7 y; *n* = 100; 52% women)	Resistance-based exercise ± leucine-enriched whey protein supplementation (0.5 g/kg/meal, 3×/day)	16 weeks	LDL ↓, insulin ↓, insulin resistance ↓ (HOMA-IR), resistin ↓ (protein only), kidney function ↔, other markers ↔	LDL ↓ (EP *p* = 0.002; P *p* = 0.003); insulin ↓ (*p* = 0.001–0.009); HOMA-IR ↓ (*p* = 0.007–0.048); eGFR unchanged	(25)
Community-dwelling pre-frail and frail older adults (mean age 73.3 ± 6.85 y; *n* = 70)	Multi-component exercise 3×/week + whey-based (2 × 20 g/day) or rice-based protein	6 months	Gait speed ↑, strength ↑, physical performance ↑, frailty ↓, muscle mass ↑; protein type effect ↔; GI symptoms ↑ (whey)	No between-group differences (*p* > 0.05); dietary intake differences only	(36)
Healthy older adults >65 y (*n* = 208)	1-year protein supplementation: carbohydrate, collagen, whey ± light-intensity or heavy-load resistance training	12 months	Protein alone ↔ (muscle mass, strength, function), heavy RT + protein: muscle size ↑, strength ↑, power ↑; light RT: strength ↑ only	HRTW ↑ qCSA (P = 0.03) & strength (*p* < 10^−4^–10^−5^); no effect of protein alone	(37)
Obese elderly HFpEF patients, grade 1–2 diastolic dysfunction (*n* = 23)	Whey protein supplementation (1.2 g/kg/day) ± light exercise (2 hydrotherapy + 1 gym session/week)	12 weeks	Protein alone ↔ (function), protein + exercise: walking capacity ↑, gait speed ↑, strength ↑ (partial improvements)	Significant improvements only in combined group (selected functional outcomes)	(26)
Sedentary older men, free-living (*n* = 32, mean age 77.4 ± 2.8 y)	Home-based resistance band training 3×/week + Muscle5 supplement (whey, micellar casein, creatine, vitamin D, omega-3) or placebo	12 weeks	Lean mass ↑, strength ↑, function ↑, muscle quality ↑, fiber size ↑ (M5), placebo ↔/↓ relative	ASM + 3%, strength (leg press) + 17%, function ↑ (p < 0.05); greater gains in sarcopenic subgroup	(27)
Older men, 69 ± 7 y, BMI 18–30 kg/m^2^ (*n* = 60)	Mixed power training (MPT) 3×/week + fast-digested protein (whey) or slow-digested protein (casein) or placebo (30 g/day)	12 weeks	Lean mass ↑, strength ↑, muscle quality ↑, function ↑ (all groups), protein type effect ↔, added protein effect ↔	Significant improvements over time (*p* < 0.05); no between-group differences	(38)
Healthy older women, 69 ± 3 y (*n* = 22)	Whey protein (WP) 30 g × 2/d or collagen protein (CP) + unilateral RE twice in 6 d	6 days	MPS ↑ (whey, rest & exercise), MPS ↑ (collagen, exercise only), long-term MPS ↑ (whey), MPS ↔ (collagen), whey > collagen ↑	Whey > collagen (P = 0.02–<0.001); significant MPS increases with whey (P < 0.01)	(42)
Community-dwelling older adults, 69 ± 6 y, 52% women (*n* = 100)	Exercise 16 wks [RE 2×/wk. + functional 1×/wk] ± leucine-enriched whey protein 1.5 g/kg/d	16 weeks	Muscle fatigue ↓, QoL ↑ (exercise), muscle mass ↔, fat mass ↔, protein effect ↔	Fatigue ↓ (P < 0.05); QoL ↑ (exercise only); no added protein effect	(39)
Older adults, >74 y, 68% women, *n* = 218	Whey-enriched protein 20 g × 2/d ± low-intensity home-based exercise	12 months + 43-month follow-up	Physical performance ↔, grip strength ↓, muscle mass ↔, functional decline ↔ (no attenuation), adverse effects ↑	SPPB change NS (*p* = 0.17); no group differences (*p* = 0.76); grip strength ↓ in all groups; GI symptoms ↑ (56%)	(41)
Post-hospitalized elderly, *n* = 28	Whey protein ± resistance training (2×/wk)	12 weeks	Physical function ↑ (both groups), muscle mass ↔, protein effect ↔ (no added benefit)	Function ↑ (*p* < 0.01); no between-group differences (*p* > 0.05)	(28)
Elderly men, 67.9 ± 0.9 y, *n* = 23	Resistance exercise + whey protein (0, 20, 40 g) post-exercise	Acute (pre, 2 h, 4 h post-exercise)	miRNA expression ↔/modulated (dose-specific), anabolic signaling ↑ (exercise-related), protein dose effect ↔ on most markers	miR-16-5p altered (*p* = 0.032); limited dose-dependent miRNA changes; signaling correlations observed	(45)
Healthy older men, 73 ± 6 y, *n* = 49	Phase 1: SUPP/CON (multi-ingredient supplement: whey 30 g, n-3 PUFA, creatine, Vit D, Ca) 20 weeks; Phase 2: RET + HIIT + continued beverages	20 weeks (12 weeks exercise + 8 weeks prior supplementation)	Cognitive function ↑ (MOCA, memory, reaction time after exercise phase), n-3 index ↑, ARA/EPA ratio ↓, composite cognition ↑ (SUPP), exercise alone ↔/↑	MOCA P = 0.013; RAVLT *p* = 0.047; reaction time *p* = 0.002; n-3 index correlated with cognition (*r* = 0.49)	(49)
Community-dwelling older adults ≥60 y, 68 ± 5 y, *n* = 46	RE 2×/wk. + FE 1×/wk. ± leucine-enriched whey protein 1.5 g/kg/day	16 weeks	Strength ↑, physical function ↑, aerobic capacity ↑ (both groups), cardiometabolic markers ↔, protein effect ↔	Strength ↑ (*P* < 0.01–<0.001); no between-group differences (*p* > 0.05)	(40)
Pre-conditioned older women, ≥60 y, *n* = 70	RT 3×/wk. + whey protein 35 g pre- or post-exercise or placebo	12 weeks	Oxidative stress ↓ (all groups), antioxidant enzymes ↑ (all groups), uric acid ↓ (whey groups), timing effect ↔	Uric acid interaction *P* < 0.001; all groups improved over time (*p* < 0.05)	(47)
Pre-conditioned older women, *n* = 70	RT 3×/wk. + whey protein 35 g pre- or post-exercise or placebo	12 weeks	Lean soft tissue ↑, intracellular water ↑, phase angle ↑, ECW/ICW ratio ↓, resistance ↔/↓ (WP groups), overall cellular health ↑ (RT + WP)	LST, ICW, ECW/ICW interaction p < 0.05; TBW, Xc, PhA time effects p < 0.05	(48)
Healthy older women, 68.8 ± 4.3 y, *n* = 30	RT 3×/wk. + whey protein 35 g vs. placebo	12 weeks	Muscle mass ↑, strength ↑, % body fat ↓, waist circumference ↓, MetS risk ↓, metabolic markers ↑ (both groups, greater in protein group)	Greater improvements in LST, WC, MetS Z-score, and body fat in protein group (*p* < 0.05)	(29)

RE, resistance exercise; RT, resistance training; MPT, mixed power training; HBRE, home-based resistance exercise; MIS, multi-ingredient supplementation; WP, whey protein; CP, collagen protein; PROT, protein; PLA, placebo; F-PROT, fast-digested protein; S-PROT, slow-digested protein; EP, exercise + protein; E, exercise only; P, protein only; CON, control; LITW, light-intensity training + whey; HRTW, heavy resistance training + whey; MIVC, maximal isometric voluntary contraction; MT, muscle thickness; MQ, muscle quality; FM, fat mass; FFM, fat-free mass; ASM, appendicular skeletal muscle mass; TLM, total lean mass; PhA, phase angle; ICW, intracellular water; ECW, extracellular water; TBW, total body water; SPPB, Short Physical Performance Battery; 1RM, one-repetition maximum; RMR, resting metabolic rate; SMR, sleeping metabolic rate; HR-QOL, health-related quality of life; AMPK, adenosine monophosphate-activated protein kinase; CRP, C-reactive protein; IL-6, interleukin-6; TNF-α, tumor necrosis factor-alpha; Bax/Bcl-2, apoptotic regulators; HOMA-IR, homeostatic model assessment for insulin resistance; LDL, low-density lipoprotein; HDL, high-density lipoprotein; RAVLT, Rey Auditory Verbal Learning Test; MOCA, Montreal Cognitive Assessment; ES, effect size; Δ, change; qCSA, quadriceps cross-sectional area. ↑ increase, ↓ decrease, ↔ no change/neutral effect.

In contrast, several randomized controlled trials conducted in healthy older adults reported limited or no additional benefits of whey protein supplementation beyond those induced by exercise alone (31–40). Although resistance exercise consistently improved muscle strength, neuromuscular performance, gait speed, functional capacity, and muscle quality, supplemental whey protein frequently failed to produce further improvements in these outcomes. Long-term studies likewise demonstrated that protein supplementation alone was generally insufficient to induce meaningful changes in muscle function or physical performance (32, 37, 41). Collectively, these findings suggest that adequate habitual protein intake and preserved anabolic responsiveness may attenuate the need for additional supplementation in healthy aging populations.

The heterogeneity of findings across studies may partly be explained by differences in protein quality and anabolic responsiveness. Mechanistic investigations consistently demonstrated that whey protein stimulates postprandial MPS more effectively than lower-quality protein sources such as collagen protein because of its higher leucine and essential amino acid content (22, 42). Acute studies further demonstrated dose-dependent stimulation of MPS following whey protein ingestion (43), whereas prolonged supplementation did not always translate into sustained increases in basal or postprandial MPS (32). Similarly, exercise-induced increases in anabolic signaling pathways and MPS were not consistently accompanied by proportional improvements in muscle strength or functional outcomes (32, 42–44). These observations suggest that acute molecular responses do not necessarily predict long-term clinical adaptation.

Emerging molecular evidence also indicates that whey protein and exercise may influence skeletal muscle adaptation through pathways extending beyond conventional anabolic signaling. Alterations in muscle-specific microRNAs involved in protein synthesis and muscle remodeling have been reported following resistance exercise and whey protein ingestion (45). Experimental evidence additionally suggests that combined nutritional and exercise interventions may modulate inflammatory pathways, oxidative stress, apoptosis, and antioxidant defense systems, thereby contributing to improved muscle function and tissue resilience during aging (46, 47). Improvements in intracellular hydration status, lean soft tissue mass, and bioelectrical markers of muscle quality have also been observed following whey protein supplementation combined with resistance training (48).

Although skeletal muscle outcomes were the primary focus of most investigations, several studies reported broader physiological effects extending beyond muscle tissue. Whey protein supplementation was associated with modest improvements in executive function and cognitive performance in some cohorts (9, 49), whereas exercise-induced reductions in systemic inflammation were not consistently correlated with cognitive outcomes (9). Other studies demonstrated improvements in health-related quality of life, fatigue resistance, neuromuscular performance, and functional independence following exercise interventions, regardless of protein supplementation status (39–41). These findings reinforce the concept that exercise exerts multisystem benefits, while the contribution of whey protein may vary according to the outcome assessed.

Overall, the collective evidence suggests that resistance exercise remains the most consistent intervention for improving muscle strength, neuromuscular function, physical performance, and muscle quality in older adults. Whey protein supplementation appears most beneficial in individuals presenting with sarcopenia, frailty, obesity, metabolic dysfunction, inadequate protein intake, or heightened anabolic resistance (22, 23, 25–29). In contrast, among healthy older adults with adequate dietary protein intake, supplementation often provides limited additional benefit beyond exercise alone (31–37, 39–41). These observations highlight the importance of individualized nutritional strategies and may explain the contradictory findings reported across clinical trials.

## 4 Whey protein–exercise interactions and anabolic resistance

### 4.1 Protein dose, quality, and digestion kinetics

Aging is correlated with anabolic resistance, a physiological state characterized by a diminished response of MPS to the intake of dietary protein and physical exercise. Whey protein is regarded as one of the most efficacious protein sources for mitigating anabolic resistance in the elderly, owing to its elevated biological value, substantial leucine content, and rapid absorption kinetics (50, 51). Leucine is crucial for the activation of the mTORC1 signaling pathway, which is integral to MPS and metabolic homeostasis (52). Research indicates that older adults necessitate higher per-meal protein quantities (25–40 g of whey protein) to achieve maximal stimulation of MPS in comparison to their younger counterparts (16, 53). The rapid absorption of whey protein leads to a transient rise in plasma amino acid availability, particularly leucine, which may partially compensate for age-related impairments in amino acid sensing and anabolic signaling. Beyond skeletal muscle, amino acids and bioactive peptides derived from whey protein have been hypothesized to influence brain function through pathways involving insulin signaling, neurotransmitter synthesis, and systemic inflammation; however, the extent of these effects in humans remains less well established, supporting a tentative view of a muscle–brain–metabolism interaction (54, 55).

### 4.2 Resistance vs. light or mixed exercise paradigms

The interplay between whey protein supplementation and exercise modality serves as a pivotal factor influencing anabolic and functional outcomes in the aging population. Conversely, light-intensity or aerobic exercise conducted in isolation elicits only modest anabolic signaling; however, it may promote mitochondrial biogenesis, enhance insulin sensitivity, and improve cerebral blood circulation. When integrated with whey protein, mixed exercise regimens may provide broader systemic benefits across physical and metabolic domains, although evidence regarding cognitive outcomes remains limited and less consistent (56, 57). Nevertheless, the extant empirical evidence concerning direct cognitive enhancements, neuroprotective implications, and exercise-induced advancements in cerebral hemodynamics in human subjects remains comparatively scarce and heterogeneous (12, 21, 58). The majority of current findings originate from limited-scale clinical trials, indirect physiological indicators, or preclinical research, thus necessitating a prudent interpretation of these prospective brain-related ramifications (13, 21). Furthermore, although aerobic and multicomponent exercise regimens have been correlated with enhancements in cognitive performance and cerebrovascular metrics in certain studies, the distinct impact of concurrent whey protein supplementation on these results has yet to be unequivocally delineated within older demographics (13, 58).

### 4.3 Evidence for synergy vs. non-additive effects

While a plethora of research elucidates the synergistic interactions between whey protein supplementation and RT regarding muscle mass and strength enhancement, the evidence indicates that these outcomes are contingent upon specific contextual factors. In individuals with sufficient protein intake or those already adhering to an adequate daily protein regimen, the addition of whey supplementation may yield non-additive or marginal enhancements (56, 59). Conversely, in older adults with inadequate protein intake, sarcopenia, or metabolic dysregulation, the combination of whey protein and exercise may be associated with more pronounced improvements in muscle hypertrophy, physical performance, and certain inflammatory and metabolic biomarkers, although the magnitude of these effects varies across studies (60, 61). Emerging evidence also suggests possible interactions at the neurobiological level, where exercise-induced myokines and amino acids derived from whey protein may interact to influence neuroplasticity, oxidative stress, and cognitive function. However, human evidence in this area remains limited and largely indirect, and therefore requires further investigation before firm conclusions can be drawn (62, 63).

## 5 Molecular and cellular biomarker modulation

### 5.1 MPS and anabolic signaling pathways

**Figure 2:**
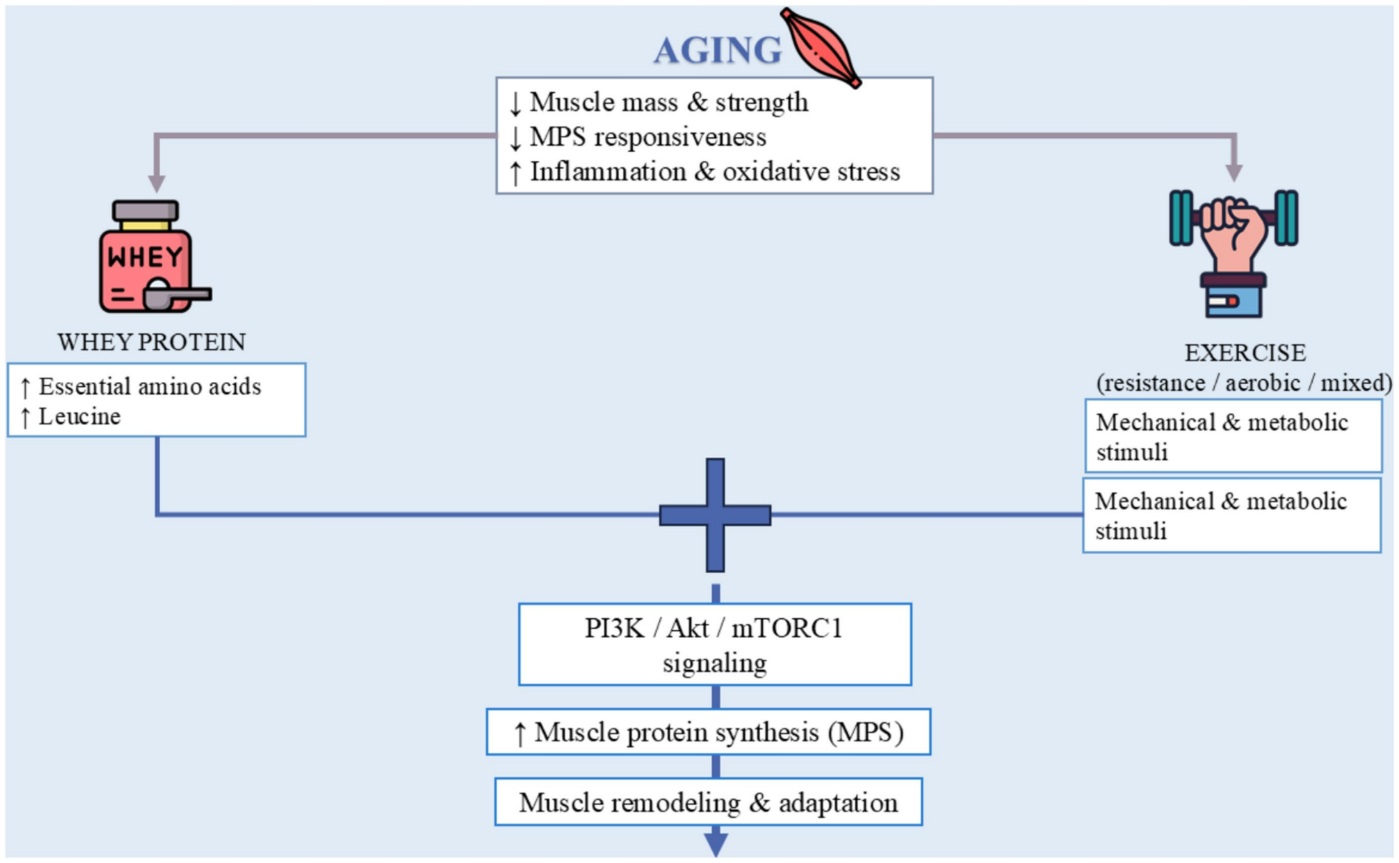
Integrated effects of whey protein and exercise on skeletal muscle health during aging. This figure summarizes how whey protein supplementation and exercise interact to counteract age-related sarcopenia and anabolic resistance. Aging is associated with reduced muscle protein synthesis (MPS), impaired mTORC1 signaling, and increased inflammation and oxidative stress. Whey protein, through rapid amino acid and leucine availability, stimulates PI3K/Akt/mTORC1 signaling, while exercise enhances anabolic sensitivity. Their convergence promotes muscle remodeling, leading to improvements in muscle mass, strength, fatigue resistance, and functional performance. At the molecular level, combined interventions reduce oxidative stress and inflammation, modulate muscle-related microRNAs, and improve cellular hydration, thereby enhancing muscle quality. The magnitude of these effects is context-dependent and influenced by baseline protein intake, exercise modality, and metabolic health.

The synergistic interactions between whey protein supplementation and physical exercise in the context of aging skeletal muscle are fundamentally governed by the modulation of MPS and anabolic signaling pathways (Figure 2). At the core of this mechanism is the PI3K/Akt/mTORC1 signaling cascade, which orchestrates the processes of translational initiation and elongation through downstream effectors, including p70S6 kinase (p70S6K) and eukaryotic initiation factor 4E-binding protein 1 (64, 65). Whey protein, characterized by rapid digestibility and high leucine content, may stimulate mTORC1 activation, particularly when consumed in close temporal proximity to RE. In older adults, this combined approach may partially attenuate anabolic resistance by influencing amino acid availability, Akt phosphorylation, and ribosomal biogenesis (66, 67). To enhance precision in the discourse, it is imperative to acknowledge that leucine functions as a pivotal anabolic amino acid that directly activates mTORC1 activity, whereas RT augments muscular sensitivity to amino acids and facilitates protein synthesis. Consequently, the synergistic effects of whey protein and RT may collaboratively support the preservation of muscle and the enhancement of functional capacity during the aging process (15, 18). Furthermore, RT enhances these effects by augmenting muscle insulin sensitivity and increasing the expression of amino acid transporters, which facilitates the availability of intracellular leucine (68). Beyond muscle hypertrophy, anabolic signaling pathways have broader systemic associations. mTORC1-related pathways have been implicated in mitochondrial function, glucose metabolism, and myokine secretion, thereby providing a potential link between muscle adaptation and systemic metabolic and neural processes within the proposed muscle–brain–metabolism axis (69).

### 5.2 Oxidative stress, inflammation, and antioxidant responses

Aging is intrinsically linked to persistent low-grade inflammation, termed “inflammaging,” as well as heightened oxidative stress, both of which detrimentally affect muscle quality, recovery, and neuromuscular functionality. The integration of exercise training with whey protein supplementation has been demonstrated to beneficially influence redox homeostasis and inflammatory markers, encompassing decreases in C-reactive protein (CRP), tumor necrosis factor-*α*, and interleukin-6 (IL-6) during resting states (60, 70). Whey protein encompasses bioactive constituents, particularly cysteine-rich peptides, which facilitate the biosynthesis of glutathione and upregulate intrinsic antioxidant mechanisms, such as superoxide dismutase (SOD), catalase, and glutathione peroxidase (71). When combined with exercise-induced activation of Nrf2 signaling pathways, whey protein supplementation may enhance antioxidant defense responses and reduce markers of oxidative stress in aging muscle (72). Significantly, appropriate regulation of oxidative stress is considered essential for adaptive training responses. Excessive antioxidant supplementation may blunt exercise-induced adaptations; however, whey protein appears to support redox balance without interfering with exercise-induced signaling, distinguishing it from high-dose antioxidant supplementation strategies (47).

### 5.3 miRNAs, cellular hydration, and muscle quality markers

Emerging empirical evidence underscores the significance of microRNAs (miRNAs) as sensitive molecular biomarkers pertinent to muscle adaptation, senescence, and the interplay between exercise and nutritional factors. Exercise alongside protein consumption modulates the expression of muscle-specific miRNAs (myomiRs), which play a pivotal role in regulating myogenesis, the activation of satellite cells, and the turnover of proteins (73, 74). Supplementation with whey protein may influence the miRNA-mediated regulation of anabolic and inflammatory signaling pathways, particularly those miRNAs that target mTORC1, FoxO, and NF-κB signaling cascades. Although the data derived from human studies remain limited, preliminary findings indicate that a combination of RE and protein intake may rectify the age-associated dysregulation of both circulating and muscle-derived miRNAs (75, 76). Furthermore, cellular hydration status emerges as another significant marker indicative of muscle quality. An increase in intracellular water content serves as an anabolic signal, thereby facilitating protein synthesis while concurrently inhibiting proteolytic processes. Whey protein intake, potentially through insulin-mediated osmotic effects and amino acid transport, may contribute to changes in cellular hydration when combined with exercise, thereby supporting muscle quality rather than solely increasing muscle mass (77, 78). Overall, miRNA profiles, hydration-related changes, and other muscle quality markers provide emerging but still developing perspectives on how the interaction between whey protein and exercise may influence muscle function and systemic health in aging populations.

### 5.4 Preclinical and clinical evidence about molecular and cellular biomarker modulation

Collectively, the available evidence indicates that the molecular and cellular responses to whey protein supplementation and exercise are highly dependent on participant characteristics, protein quality, anabolic sensitivity, and the biological endpoint assessed. While several studies demonstrated favorable effects on body composition, muscle quality, and cardiometabolic biomarkers, the magnitude of these effects varied substantially across populations and intervention protocols.

Clinical studies generally suggest that whey protein supplementation may enhance selected body composition and metabolic outcomes when combined with resistance training. Improvements in appendicular lean soft tissue, skeletal muscle mass, muscle strength, lipid profiles, training volume, and functional performance have been reported in older adults receiving whey protein supplementation alongside resistance exercise (79–82) (Table 2). Additional benefits have also been observed in mobility-limited or vitamin D-deficient older adults, including reductions in intermuscular adipose tissue, improvements in muscle density, and enhanced preservation of physiological status when nutritional support was combined with exercise interventions (83, 84). However, several investigations reported that although exercise consistently improved gait speed, physical performance, muscle strength, and body composition, supplementation provided limited or no additional benefit beyond exercise alone (85, 86). These findings suggest that the clinical efficacy of whey protein may depend on baseline nutritional status, anabolic resistance, mobility limitations, and metabolic health.

**Table 2:** Effects of whey protein supplementation combined with resistance or aerobic exercise on molecular and cellular biomarker modulation in older adults.

Population/Sample size	Intervention	Duration and training protocol	Outcomes	Key statistical results	Reference
70 pre-conditioned older women	Whey protein (35 g) pre-RT or post-RT vs. placebo	12 weeks; RT 3×/week; 3 sets of 8–12 RM	Lean mass ↑, ALST ↑, fat mass ↓ (post-RT WP strongest), TC/HDL ratio ↓, metabolic profile ↑, inflammation ↔/↓	ALST ↑ (*P* < 0.05); TC/HDL ↓ (*p* < 0.05); post-RT WP most effective for fat loss	(79)
32 pre-conditioned older women	Whey protein (35 g post-RT) vs. placebo	12 weeks; RT 3×/week; 3 sets of 8–12 reps	Lean mass ↑, load volume ↑, TC/HDL ratio ↓, lipid profile ↑, inflammatory markers ↓ (both groups), waist circumference ↔	Group × time: LST *p* < 0.05; TC/HDL *p* < 0.05; HDL, LDL, TG improved in both groups	(80)
70 pre-conditioned older women	Whey protein (35 g) pre-RT or post-RT vs. placebo	12 weeks; RT 3×/week; 3 sets of 8–12 RM	Muscle mass ↑, strength ↑, functional capacity ↑ (all WP groups), timing effect ↔, placebo smaller improvements ↑	SMM ↑ (*p* < 0.05); strength ↑ (*p* < 0.05); 10-m walk improved (p < 0.05); no timing differences	(81)
27 older men (69 ± 1 y)	Whey hydrolysate or caseinate vs. carbohydrate, with unilateral heavy resistance exercise	Acute study; 10 × 8 reps at 70% 1RM	MPS ↑ (feeding all groups); Exercise →; p70S6K →	No group differences in FSR; fed states ↑ vs. basal (*p* < 0.001); resistance exercise → no additional MPS effect	(92)
31 preconditioned older women (67.4 ± 4.0 y)	Whey protein (35 g/day) vs. placebo combined with RT	12 weeks; RT 3×/week	Muscle mass ↑; Strength ↑; Muscle quality ↑ (both groups, no difference)	WP vs. PLA: mass ↑ (+4.8% vs. + 2.3%, *p* < 0.05); strength ↑ (+8.7% vs. + 4.9%, p < 0.05); muscle quality → (between groups)	(82)
149 mobility-limited older adults (77.5 ± 5.4 y)	Nutritional supplement (20 g whey protein + vitamin D) vs. placebo plus structured physical activity	24 weeks; PA 3×/week (walking, strength, balance)	Gait speed ↑ (both groups); Physical function →; Vitamin D ↑	No between-group differences in gait speed (*p* = 0.06); SPPB →; vitamin D ↑ in supplement group only	(85)
24 older women (65 ± 1 y)	Leucine-enriched EAA (1.5 g or 6 g) vs. whey protein (40 g), at rest and with exercise	Acute study; unilateral resistance exercise (6 × 8 reps at 75% 1RM)	MPS ↑ (feeding); MPS + exercise ↑; p70S6K ↑ (WP only); blood flow →	FED ↑ MPS in all groups; FED-EX ↑ MPS in all groups (*p* < 0.05); no clear dose–response; WP and LEAA_6 only ↑ MPS above basal over 0–4 h	(87)
149 mobility-limited older adults (78.5 ± 5.4 y; 46% female)	Whey protein (20 g) + vitamin D vs. placebo combined with physical activity	6 months; PA including walking, strength, balance	Muscle density ↑; Intermuscular fat ↓; Strength ↑ (both groups); Overall body composition ↑	Supplement group had greater ↓ intermuscular fat (*p* = 0.049) and ↑ muscle density (*p* = 0.018); both groups improved significantly	(83)
34 elderly patients (66.5, mixed sex)	Resistance training alone vs. resistance training + whey protein and vitamin D	3 months; resistance training	Muscle strength ↑ (RT + nutrition); Muscle mass → (RT only); Sarcopenia status ↓ (trend)	Combined intervention increased strength significantly (*p* = 0.027); RT alone → no significant change	(84)
Aged rats (17–19 months)	Casein, whey, or soluble milk protein with or without low-intensity physical activity	2 months; treadmill low-intensity PA	Locomotion ↑ (soluble milk protein + exercise); Muscle mass → (no major change); Strength ↑ (functional only)	Soluble milk protein + exercise improved gait and locomotor activity more than casein/whey; muscle mass differences → minimal	(94)
16 older women (~66 ± 2.5 y)	Low-dose leucine-enriched EAA (3 g; 40% leucine) vs. whey protein (20 g), at rest and with exercise	Acute study; unilateral resistance exercise (6 × 8 reps at 75% 1RM)	MPS ↑ (feeding); MPS + exercise ↑↑; APS ↑; signaling ↑ (p70S6K after exercise)	LEAA ≈ whey for MPS stimulation; no difference between groups (*p* > 0.05 for most comparisons)	(88)
19 healthy older adults	High whey protein, leucine-enriched supplement vs. iso-caloric dairy control post-exercise	Acute study; unilateral resistance exercise	MPS ↑ (higher in whey/leucine group); Exercise × supplement →; Insulin →	EXP group higher FSR (0.078 vs. 0.057%/h, *p* = 0.049); no interaction with exercise	(89)
80 mobility-limited older adults (70–85 y)	Whey protein concentrate (40 g/day) vs. isocaloric control combined with RT	6 months; progressive high-intensity RT	Lean mass ↑ (small); Strength ↑ (both groups); Function ↑ (both groups); Between-group effect →	No significant differences between whey vs. control for any outcome (all *p* > 0.05)	(86)
37 elderly men (71 ± 4 y)	Whey protein isolate 0, 10, 20, 40 g, at rest and post unilateral resistance exercise	Acute study; unilateral leg resistance exercise	MPS ↑; leucine oxidation ↑; dose–response effect	MPS ↑ ~ 65% (W20) and ↑ ~ 90% (W40) vs. fasting (*p* < 0.05); W20 and W40 > W0/W10; W40 > W20; leucine oxidation ↑	(90)
14 elderly men (72 ± 1 y)	Whey protein isolate (20 g) vs. micellar casein (20 g), at rest and post unilateral resistance exercise	Acute study; unilateral leg resistance exercise	MPS ↑ (whey > casein) at rest and post-exercise	Rest MPS ↑ whey vs. casein (*P* = 0.002); exercise MPS ↑ whey vs. casein (*p* < 0.001)	(91)
24 elderly men and women (68 ± 1 y)	Whey protein vs. caseinate, pre- or post-resistance exercise vs. control	Acute study; heavy resistance exercise	MPS ↔ (whey vs. casein); MPS ↔ (pre vs. post timing)	No significant differences; MPS similar across all protein timing and types	(93)
12 older adults (59 ± 4 y)	Protein + carbohydrate (PRO) vs. carbohydrate alone (CHO) immediately after aerobic exercise	Acute study; 1 h aerobic exercise at ~50% VO₂max	Whole-body protein turnover ↑; leucine appearance ↑; oxidation ↑; protein use ↑	PRO > CHO (P = 0.001): leucine appearance ↑, oxidation ↑, non-oxidative disposal ↑	(95)

WP, whey protein; EAA, essential amino acids; LEAA, leucine-enriched essential amino acids; WPC, whey protein concentrate; ALST, appendicular lean soft tissue; LST, lean soft tissue; SMM, skeletal muscle mass; MPS, muscle protein synthesis; FSR, fractional synthesis rate; TC/HDL-C, total cholesterol/high-density lipoprotein cholesterol ratio; CRP, C-reactive protein; VL, training volume; RT, resistance training; PA, physical activity; SPPB, short physical performance battery; FED, feeding; FED-EX, feeding plus exercise; MBF, muscle microvascular blood flow; LBF, leg femoral blood flow; APS, albumin protein synthesis; ↔, no effect; ↑, increase; ↓, decrease.

At the molecular level, a substantial body of evidence supports the ability of whey protein to stimulate MPS and anabolic signaling pathways. Studies comparing whey protein with placebo or lower-protein control supplements consistently demonstrated greater postprandial MPS responses, particularly when protein intake was combined with resistance exercise (87–91). Dose–response investigations further revealed that higher post-exercise whey protein intakes produced greater stimulation of MPS than lower doses, suggesting that older adults may require relatively larger protein doses to maximize anabolic responsiveness (89, 90). Moreover, whey protein frequently induced greater activation of anabolic signaling mediators, including p70S6K1 phosphorylation, and produced more favorable amino acid availability profiles than control conditions (87, 91).

Protein quality emerged as another important determinant of anabolic responsiveness. Several studies demonstrated superior acute anabolic responses following whey protein ingestion compared with lower-quality or slowly digested protein sources because of differences in leucine availability and amino acid kinetics (89, 91). However, not all investigations reached similar conclusions. Some studies reported comparable rates of MPS and anabolic signaling following ingestion of whey protein, caseinate, or alternative protein formulations under specific experimental conditions (92, 93). Likewise, animal studies suggested that functional adaptations may be influenced not only by protein composition but also by digestibility characteristics and timing of administration relative to exercise (94). Collectively, these findings indicate that protein quality influences anabolic responses, although the magnitude of its effects may vary according to exercise status, feeding strategy, and participant characteristics.

An important observation across the literature is the frequent dissociation between molecular adaptations and clinical outcomes. Although whey protein consistently enhanced acute markers of anabolic activity, including MPS, amino acid availability, whole-body protein turnover, and anabolic signaling pathways (87–93, 95), these mechanistic responses did not always translate into superior long-term improvements in muscle strength, physical function, or mobility (85, 86). This discrepancy suggests that acute molecular responses represent only one component of the adaptive process and that long-term functional outcomes are influenced by multiple interacting factors, including exercise intensity, training duration, habitual dietary intake, baseline muscle status, age-related anabolic resistance, and overall health status.

Overall, current evidence supports resistance training as the primary driver of improvements in muscle function and physical performance, whereas whey protein supplementation appears to exert its greatest influence through modulation of anabolic signaling, muscle protein synthesis, protein turnover, body composition, and selected metabolic biomarkers. The heterogeneous findings across studies likely reflect differences in participant characteristics, protein quality, dosing strategies, exercise protocols, and the distinction between short-term molecular responses and long-term functional adaptations.

## 6 Cognitive function and the muscle–brain axis

An expanding body of evidence corroborates the notion of a muscle–brain–metabolism axis, wherein skeletal muscle functions as an endocrine organ that influences cerebral health through exercise-induced myokines, metabolic regulators, and inflammatory mediators.

### 6.1 Exercise-induced neurocognitive adaptations

Physical activity incites the upregulation of neurotrophic factors such as BDNF, IGF-1, and vascular endothelial growth factor, which collectively bolster neuronal viability, learning mechanisms, and memory functions (12, 96). From a muscle-brain interaction perspective, the contraction of skeletal muscle leads to the release of myokines including irisin, cathepsin B, and IL-6, which may contribute to central nervous system signaling and brain function regulation. Irisin, derived from fibronectin type III domain-containing protein 5, has been suggested to cross the blood–brain barrier and may be associated with increased hippocampal BDNF expression, potentially supporting synaptic plasticity and cognitive performance (97, 98). Furthermore, exercise-induced improvements in insulin sensitivity and mitochondrial function are believed to contribute to more efficient cerebral energy metabolism (99).

### 6.2 Role of whey protein and multi-ingredient supplementation

Whey protein is abundant in essential amino acids, most notably leucine, which initiates the mTORC1 signaling pathway and fosters muscle anabolism. The preservation of muscle mass and strength exhibits a robust association with improved cognitive outcomes among older adults, thereby underscoring the interrelation between physical and cognitive health (100). Emerging evidence suggests that whey protein supplementation may contribute to cognitive performance through modulation of oxidative stress, glucose metabolism, and chronic low-grade inflammation, which are recognized contributors to age-related cognitive decline (101, 102). The use of multi-ingredient supplementation strategies combining whey protein with omega-3 fatty acids, creatine, vitamin D, or polyphenols has been explored as a potential approach to support both physical and cognitive outcomes. These formulations target multiple biological pathways, including inflammation, mitochondrial function, and neuromuscular signaling, and are consistent with broader concepts in preventive and personalized nutrition; however, the evidence remains heterogeneous and not yet conclusive (103, 104). Therefore, findings derived from multi-nutrient interventions should be interpreted cautiously when evaluating the specific contribution of whey protein to muscle–brain axis regulation and healthy aging outcomes.

### 6.3 Links between muscle-derived signals, metabolism, and cognition

Myokines released from muscle tissue play a pivotal role in the regulation of systemic glucose and lipid metabolism, thereby affecting insulin sensitivity in the brain and the availability of energy substrates. The disruption of these signaling pathways during the aging process is implicated in the deterioration of cognitive function and an elevated susceptibility to neurodegenerative disorders (5). Nevertheless, it is imperative to underscore that the predominant body of evidence correlating physical exercise, muscle-derived signaling pathways, and cognitive or cerebrovascular enhancements in humans is chiefly attributable to adaptations induced by exercise, rather than to the supplementation of whey protein itself. Although whey protein may facilitate muscle anabolism and may indirectly enhance systemic metabolic health, its direct influence on cognitive outcomes remains inadequately substantiated and is predominantly indirect according to existing human research (21, 58). Consequently, the cognitive enhancements and improvements in cerebral blood flow evidenced in the majority of interventions are more reliably ascribed to the effects of exercise training, with nutritional supplementation functioning as a secondary or ancillary component (105).

### 6.4 Integrated effects of whey protein, exercise, and metabolic supplements on neuromuscular and neurocognitive outcomes

Collectively, evidence from randomized controlled trials suggests that the combined effects of RT and nutritional supplementation (including whey protein, creatine, and amino acid derivatives) on neuromuscular function and body composition are highly context-dependent and largely driven by exercise stimulus rather than supplementation alone.

### 6.5 Neuromuscular adaptations and functional performance: predominant role of resistance training

A consistent finding across clinical trials is that RT induces significant improvements in muscle strength, functional performance, and mobility outcomes, while supplementation often provides limited or non-additive effects. For example, in frail elderly individuals, both whey protein alone and whey protein combined with creatine significantly improved handgrip strength, timed-up-and-go performance, and sit-to-stand ability; however, no significant between-group differences were observed, indicating that RT was the primary driver of adaptation (106) (Table 3). Similarly, in non-frail older adults, cysteine-enriched whey protein (Immunocal®) induced slightly greater strength gains compared to casein, although both groups exhibited substantial improvements in muscle strength without significant changes in lean body mass (107). In postmenopausal women undergoing low-intensity high-volume RT, significant increases in muscle strength and muscle thickness were observed; however, no differences were detected between whey protein and placebo groups, again emphasizing the dominant role of exercise stimulus (108). Further supporting this pattern, creatine and/or whey protein supplementation during RT failed to produce additional benefits in strength or lean body mass compared with RT alone in middle-aged and older male cohorts (109, 110). Collectively, these findings indicate that in well-nourished or moderately active populations, RT remains the primary determinant of neuromuscular adaptation, while supplementation effects are minimal or absent.

**Table 3:** Effects of whey protein supplementation combined with exercise on cognitive function and the muscle–brain axis in older adults.

Population/Sample size	Intervention	Duration and training protocol	Outcomes	Key statistical results	References
Older adults with frailty	Whey protein + creatine vs. whey protein alone, combined with resistance training	14 weeks; supervised RT	Muscle function ↑; body composition ↔; blood markers ↔; safety ↑ (no adverse effects)	No between-group differences (*p* > 0.05); time effects: handgrip ↑, timed-up-and-go ↓ (improved), sit-to-stand ↑; creatine add-on ↔ vs. whey alone	(106)
99 non-frail elderly (84 completed; 67 high compliance analyzed)	Cysteine-rich whey protein (Immunocal®) vs. casein, combined with resistance training	135 days; 3×/week RT	Muscle strength ↑ (greater with whey); lean body mass ↔	Strength ↑ ~ 31–43% both groups; Immunocal® showed additional ↑ (~10% vs. casein, *p* < 0.05); LBM ↔ (no change)	(107)
12 postmenopausal women (57 ± 4.7 y)	Whey protein vs. placebo, combined with high-volume unilateral RT	10 weeks; 2×/week unilateral RT	Muscle strength ↑; muscle thickness ↑; lean mass ↔	Strength ↑ (*p* = 0.006); muscle thickness ↑ (*p* = 0.022); no differences between whey and placebo (whey ↔ effect vs. placebo)	(108)
42 middle-aged and older men (48–72 y)	Creatine, whey protein, creatine + whey protein, or placebo, combined with resistance training	14 weeks; 3×/week progressive RT	Muscle strength ↑; lean body mass ↑ (training effect); supplementation ↔	All groups ↑ strength and LBM (*P* < 0.05); no group differences or interaction effects ↔	(109)
42 middle-aged men (48–72 y)	Creatine, whey protein, creatine + whey protein, or placebo, combined with resistance training	14 weeks; 3×/week RT	Body composition changes ↑ (training-driven); lean mass ↑; body water ↑; fat mass ↓; supplementation ↔	Significant training effects: arm fat ↓, BF-FFM ↑, TBW/ICW/ECW ↑; no group effects ↔; only ECW interaction significant	(110)
8 elderly men (73 ± 1 y)	Carbohydrate + protein hydrolysate vs. carbohydrate + protein + leucine post-exercise	Single session, cross-over	Muscle protein synthesis ↔; protein balance ↑ (with leucine); oxidation ↓	MPS (FSR) 0.081 vs. 0.082%/h ↔ (NS); whole-body protein balance ↑ with leucine (P < 0.05); oxidation ↓	(111)

WHEY + CR, whey protein and creatine co-supplementation; WHEY, whey protein supplementation; Immunocal®, cysteine-rich whey protein isolate; LBM, lean body mass; DXA, dual-energy X-ray absorptiometry; RT, resistance training; 1RM, one-repetition maximum; SPPB, short physical performance battery; WBPT, whole-body protein turnover; PRO, protein plus carbohydrate beverage; CHO, carbohydrate beverage. ↑ increase, ↓ decrease, ↔ no change/neutral effect.

### 6.6 Muscle protein metabolism and anabolic signaling: context-dependent effects of supplementation

At the molecular level, whey protein and leucine-enriched formulations consistently stimulate muscle protein metabolism; however, the magnitude of these effects depends on protein dose, timing, and baseline nutritional status. A crossover study in older males demonstrated that leucine-enriched supplementation did not further enhance MPS or fractional synthetic rate beyond protein hydrolysate alone, although modest improvements in whole-body protein balance were observed (111). These findings suggest a ceiling effect for anabolic stimulation when adequate protein is already provided. Overall, evidence indicates that whey protein, particularly when enriched with leucine or cysteine, enhances postprandial aminoacidemia and activates anabolic signaling pathways such as p70S6K1, thereby supporting MPS during rest and post-exercise conditions. However, simultaneous supplementation with creatine or additional leucine does not consistently augment these responses when dietary protein intake is sufficient (109–111). Thus, anabolic responses appear to be highly sensitive to nutritional context rather than representing universal effects across populations.

### 6.7 From muscle to brain: neurocognitive outcomes and the muscle–brain axis

Emerging evidence suggests a potential interaction between neuromuscular adaptations and neurocognitive function; however, the current body of literature remains largely indirect, with most findings derived from systemic biomarkers or global cognitive screening instruments. In several included studies, improvements in muscle strength and metabolic health were accompanied by parallel, but non-specific, enhancements in cognitive outcomes, suggesting that any neurocognitive benefits are likely mediated through systemic metabolic, inflammatory, and vascular pathways rather than direct neuromuscular–cortical mechanisms. Importantly, the majority of human studies in the whey protein–exercise field rely on peripheral biomarkers (e.g., inflammatory and metabolic indices) and cognitive screening tools such as global cognitive scales, which limits mechanistic interpretation at the level of brain function. As a result, direct evidence linking skeletal muscle adaptations to specific neural processes underlying executive function or working memory remains insufficient. From a mechanistic neuroscience perspective, these observations can be conceptually integrated within established neuroplasticity frameworks, including cortico-hippocampal network remodeling, synaptic plasticity, and neurovascular coupling (112, 113). In this context, exercise-induced peripheral adaptations such as improved insulin sensitivity, reduced systemic inflammation, and enhanced mitochondrial efficiency, may indirectly support cerebral energy metabolism and vascular function, thereby creating a permissive environment for neuroplastic processes. Nevertheless, such mechanistic links remain largely inferential within the current whey protein–exercise literature. Furthermore, objective neurophysiological techniques capable of capturing brain dynamics with high temporal resolution, such as electroencephalography and event-related potentials, have rarely been incorporated in studies investigating whey protein and exercise interventions (114, 115). The absence of these methods limits the ability to directly quantify cortical excitability, neural efficiency, and information-processing speed associated with executive and memory functions. Consequently, evidence for brain-level adaptations remains indirect and primarily hypothesis-driven. Taken together, the current literature supports a model in which exercise represents the principal driver of neurocognitive benefits, while whey protein and related nutritional strategies may exert secondary, supportive effects through systemic metabolic optimization. However, the mechanistic pathway linking muscle adaptations to cognitive enhancement remains incompletely understood and requires future studies integrating neuroimaging and neurophysiological approaches to directly assess brain plasticity in response to combined exercise–nutrition intervention.

### 6.8 Integrated interpretation and evidence synthesis

Across the included studies, a consistent pattern emerges indicating that RT represents the primary and most robust stimulus for improving muscle strength, functional capacity, and neuromuscular performance in older adults. In contrast, nutritional supplementation strategies—including whey protein, creatine, leucine-enriched formulations, and combined multi-nutrient interventions—exert variable and largely context-dependent effects that are strongly influenced by baseline nutritional status, anabolic responsiveness, and overall health condition. Overall, the evidence suggests that supplementation tends to provide greater benefits in populations characterized by frailty, sarcopenia, metabolic impairment, or low habitual protein intake, where anabolic resistance is more pronounced. In these populations, combined RT and nutritional strategies are more likely to enhance muscle mass, strength, and functional outcomes compared with RT alone. Conversely, in healthy older adults with adequate dietary protein intake, supplementation frequently fails to produce additional improvements beyond those achieved through exercise training alone. At the mechanistic level, whey protein and related amino acid-based supplements are associated with modulation of anabolic signaling pathways involved in muscle protein metabolism and stimulation of MPS. However, despite these acute molecular responses, such effects do not consistently translate into superior long-term adaptations in muscle strength, physical performance, or functional capacity. This dissociation between molecular and functional outcomes suggests that anabolic signaling alone is insufficient to explain whole-body adaptation to training in older populations. Collectively, the evidence supports a hierarchical model of adaptation in which RT serves as the primary driver of neuromuscular and functional improvements, while nutritional supplementation acts as a modulatory factor that may enhance anabolic sensitivity under specific physiological conditions. The magnitude of supplementation benefits is therefore highly dependent on baseline nutritional adequacy, metabolic health, and the degree of anabolic resistance. While emerging literature suggests potential interactions between peripheral muscle adaptations and central nervous system function, neurocognitive mechanisms were not consistently evaluated across the included trials. Therefore, the relationship between muscle adaptations and brain-related outcomes remains indirect and requires further investigation using objective neurophysiological approaches and dedicated mechanistic studies. Overall, current evidence does not support a universal additive effect of supplementation over RT, but rather indicates a context-dependent interaction between exercise stimulus and nutritional status in determining neuromuscular outcomes in aging populations.

## 7 Clinical and functional implications in aging populations

The integrative utilization of physical exercise and whey protein supplementation presents potential clinical and functional implications for aging populations, especially in the context of frailty, sarcopenia, and cardiometabolic disorders. These pathological conditions frequently coexist and interact via shared mechanisms, including persistent low-grade inflammation, insulin resistance, mitochondrial dysfunction, and anabolic resistance, all of which may contribute to the deterioration of both physical and cognitive functions as individuals age.

### 7.1 Frailty, sarcopenia, and cardiometabolic conditions

Frailty and sarcopenia represent highly prevalent syndromes associated with aging, characterized by a decrease in muscle mass, strength, and functional capacity, which culminates in elevated risks of falls, disability, hospitalization, and mortality. Sarcopenia has been increasingly acknowledged as a fundamental biological underpinning of frailty and is intricately associated with compromised metabolic health and has been suggested to be associated with cognitive decline, although the evidence remains indirect and not fully conclusive (116, 117). Physical exercise particularly resistance and multicomponent training, constitutes the primary intervention for the prevention and management of sarcopenia. Nevertheless, anabolic resistance observed in older adults frequently attenuates the muscle protein synthetic response to exercise alone, thereby necessitating the incorporation of specific nutritional support. The supplementation of whey protein, owing to its rapid digestibility and elevated leucine concentration, effectively enhances MPS and amplifies the adaptive response to exercise in elderly populations (118). The preservation of muscle mass and strength yields consequential benefits on mobility and balance, while evidence for injury prevention remains supportive but not fully consistent across studies, and may indirectly contribute to metabolic and cognitive health through enhanced muscle–brain communication. From a cardiometabolic standpoint, sarcopenia is often found concurrently with obesity, insulin resistance, type 2 diabetes, and cardiovascular pathology—a phenomenon frequently referred to as sarcopenic obesity. Engaging in physical exercise in conjunction with whey protein supplementation has been evidenced to enhance glycemic regulation, lipid profiles, arterial pressure, and systemic inflammation, consequently mitigating cardiometabolic risk while preserving lean mass (80). These metabolic enhancements hold significant clinical implications, as cardiometabolic dysfunction is a principal factor associated with cognitive decline and the risk of dementia in aging populations; however, a direct causal link between whey protein supplementation and cognitive protection remains insufficiently established.

### 7.2 Tolerability, adherence, and GI considerations

Notwithstanding the established advantages associated with protein supplementation, the factors of tolerability and sustained adherence are paramount considerations among the geriatric population. The aging process is frequently correlated with alterations in appetite regulation, sensory perception of taste, dental health, and GI functionality, which may impede both dietary protein consumption and compliance with supplementation regimens. Whey protein is typically regarded as well tolerated due to its superior digestibility and beneficial amino acid composition; nonetheless, GI adverse effects such as bloating, nausea, or discomfort may manifest in individuals exhibiting lactose intolerance or diminished digestive capabilities (51). Approaches aimed at enhancing tolerability and adherence encompass the implementation of lactose-reduced or hydrolyzed whey protein formulations, distributing protein consumption across multiple meals, and incorporating supplements into familiar culinary contexts. Significantly, the integration of nutritional supplementation with structured exercise programs may support adherence and engagement in some studies, although evidence remains limited and context-dependent, thereby potentially contributing to sustained behavioral compliance in older adults (119). From a clinical application perspective, personalized dosing protocols that take into account body mass, levels of physical activity, renal functionality, and existing comorbidities are imperative.

## 8 Heterogeneity of findings and sources of inconsistency

Despite the increasing scholarly interest in the potential interactions between whey protein supplementation and physical exercise on muscle function, metabolic health, and to a lesser extent cognitive outcomes, the existing literature reveals substantial heterogeneity. The divergent results observed across various studies can primarily be attributed to discrepancies in baseline protein intake, training status, demographic variables, health-related conditions, as well as variations in intervention duration and participant adherence. Comprehending these sources of variability is crucial for the accurate interpretation of findings and for the development of personalized approaches within the muscle–brain–metabolism framework. However, current evidence supports more consistent effects on muscle-related outcomes compared to cognitive or injury-related endpoints.

### 8.1 Baseline protein intake and training status

One significant factor contributing to the discrepancies observed in research findings is the variability in baseline dietary protein intake and habitual physical activity levels among the participants involved in the studies. Older adults who demonstrate adequate or elevated baseline protein consumption may exhibit diminished responses to whey protein supplementation, potentially attributable to a ceiling effect on MPS and associated metabolic pathways. Conversely, individuals with inadequate protein intake are likely to show more pronounced enhancements in muscle mass, strength, and metabolic biomarkers subsequent to supplementation. Similarly, training status exerts an important modulatory influence. Exercise-naïve older adults frequently experience greater relative improvements in physical performance and neuromuscular adaptations, along with changes in exercise-induced myokine signaling, which have been hypothesized to contribute to muscle–brain communication, although direct evidence for cognitive benefits remains limited. In contrast, older individuals who are physically active or resistance-trained may require higher protein dosages, optimized timing (e.g., post-exercise intake rich in leucine), or longer intervention durations to achieve additional benefits. The failure to appropriately stratify participants based on initial nutritional status and training experience likely contributes to, but does not fully explain, the heterogeneous results observed across studies.

### 8.2 Age, sex, health status, and exercise modality differences

Age-related biological variability further complicates the interpretation of outcomes in aging research. Younger-old adults (approximately 60–70 years) typically exhibit a higher degree of anabolic sensitivity to both protein consumption and exercise stimuli in comparison to the oldest-old population (>75–80 years), who may present with anabolic resistance, persistent low-grade inflammation, mitochondrial dysfunction, impaired recovery capacity, and compromised neuromuscular signaling (116). These physiological alterations may attenuate, rather than uniformly suppress, the potential synergistic effects of whey protein supplementation and exercise on muscle–brain communication and systemic metabolism. Sex-specific differences remain insufficiently explored within the current literature. Fluctuations in hormonal levels, particularly age-associated reductions in estrogen and testosterone, substantially influence muscle metabolism, inflammatory regulation, vascular function, and neuroplasticity (120). Consequently, men and women may exhibit distinct responses to integrated nutritional and exercise interventions targeting the muscle–brain–metabolism axis. However, current evidence remains inconclusive because many studies are underpowered to detect sex-specific effects or fail to stratify outcomes according to sex. In addition, variability in health status markedly influences responsiveness to intervention strategies. The presence of chronic diseases, sarcopenia, frailty, insulin resistance, cardiovascular dysfunction, or polypharmacy may alter protein digestion, amino acid bioavailability, mitochondrial adaptations, exercise tolerance, and biomarker responses, thereby contributing to inconsistency across aging cohorts. These factors may also influence neurocognitive responsiveness through mechanisms involving chronic inflammation, oxidative stress, cerebrovascular dysfunction, and altered neurotrophic signaling. Importantly, exercise modality and intensity represent additional major sources of heterogeneity. Moderate-intensity continuous training may preferentially improve aerobic metabolism, lipid utilization, and cardiovascular efficiency, whereas HIIT has been more strongly associated with improvements in mitochondrial biogenesis, insulin sensitivity, vascular responsiveness, and neurotrophic signaling pathways such as BDNF expression (121). These physiological differences may differentially influence executive function, cognitive flexibility, and metabolic adaptations associated with the muscle–brain axis. Nevertheless, tolerance to higher-intensity exercise may vary considerably among older adults depending on baseline fitness, frailty status, and comorbidities. Moreover, intervention strategies may require specific adaptation for clinically vulnerable populations, particularly older adults with mild cognitive impairment or elevated risk of neurodegenerative diseases. Emerging evidence suggests that multimodal interventions combining resistance exercise, aerobic training, balance exercises, and cognitively engaging motor activities may provide superior benefits for neurocognitive preservation compared with single-modality interventions (122). In such populations, individualized progression of exercise intensity, greater supervision, optimized protein timing and leucine availability, and longer intervention durations may be necessary to effectively target both neuromuscular and neurocognitive pathways while maintaining safety and adherence. Collectively, these multiple sources of heterogeneity indicate that the efficacy of combined whey protein supplementation and exercise interventions is highly individualized and should be interpreted within the context of baseline physiological reserve, metabolic status, cognitive function, and clinical vulnerability.

### 8.3 Intervention duration and compliance

Variation in the duration of interventions represents a significant source of inconsistency within the research domain. Short-term investigations (≤8–12 weeks) may effectively capture initial enhancements in MPS or alterations in circulating biomarkers; however, they often fall short in identifying substantial changes in cognitive functionality, injury susceptibility, or long-term metabolic adaptations. Conversely, extended interventions are more likely to uncover enduring modifications within the muscle–brain–metabolism interplay, encompassing neurotrophic signaling pathways, enhanced insulin sensitivity, and a decrease in systemic inflammatory responses. Nevertheless, longer study durations do not consistently guarantee superior outcomes, as results remain influenced by multiple interacting factors. Compliance among participants exerts a considerable impact on the outcomes of studies. Adherence to protocols surrounding protein supplementation, exercise intensity, frequency, and progression frequently remains suboptimal within older demographics, attributable to challenges such as gastrointestinal tolerance, musculoskeletal constraints, or motivational obstacles. Insufficient documentation or monitoring of compliance hinders comparability across studies and may obscure the true synergistic effects of combined interventions. Therefore, variability in adherence should be considered a critical confounding factor when interpreting reported efficacy.

## 9 Future directions and knowledge gaps

Despite the increasing scholarly interest in the synergistic effects of whey protein supplementation coupled with exercise interventions to promote healthy aging, several significant deficiencies persist that ought to inform forthcoming research endeavors. Current empirical evidence elucidates considerable inter-individual variability in the physiological responses to the combination of whey protein supplementation and exercise, thereby accentuating the necessity for precision-oriented methodologies. However, the extent of this variability and its underlying determinants remain incompletely understood. Subsequent investigations should categorize participants based on their baseline protein intake, anabolic sensitivity, sex, age, metabolic health, and functional status. It will be imperative to optimize factors such as protein dosage, timing, amino acid profile, and digestive kinetics in conjunction with particular exercise modalities and intensities to better understand their potential to achieve maximal functional and cognitive benefits. The incorporation of genetic, epigenetic, and metabolomic analyses may provide additional insights into the specificity of individualized nutrition-exercise regimens for older adults. The nascent concept of the muscle-brain-metabolism axis presents an innovative framework for comprehending how peripheral muscular adaptations may influence central nervous system functionality throughout the aging process. Future investigations should clarify the contributions of muscle-derived factors and metabolic intermediates in mediating cognitive enhancements and neuroprotective outcomes. At present, evidence supporting these pathways is still emerging and not yet fully conclusive. Investigating the interplay between whey protein and physical exercise in modulating myokines, insulin sensitivity, lipid metabolism, and neurotrophic signaling may help inform the formulation of therapeutic strategies that extend beyond musculoskeletal health to include cognitive function and metabolic resilience. The majority of existing studies are constrained by relatively brief intervention periods, limited sample sizes, and a predominant emphasis on functional outcomes. There is a necessity for long-term RCTs with sufficient statistical power to determine whether the combination of whey protein and exercise interventions can sustainably modify the trajectories of physical decline, cognitive deterioration, and frailty in aging populations. Concurrently, mechanistic studies involving human subjects that incorporate muscle and blood biomarkers, advanced imaging techniques, stable isotope methodologies, and molecular analyses are essential to bridge the divide between acute biological responses and long-term clinical outcomes. Such integrative approaches are likely to be important for the translation of mechanistic insights into evidence-based recommendations for promoting healthy aging.

## 10 Conclusion

This review underscores that physical exercise remains one of the foremost and most consistent interventions for enhancing physical performance, muscle functionality, and various dimensions of cognitive health among aging populations. Resistance, power, and multimodal training regimens consistently mitigate age-associated declines, whereas whey protein supplementation in isolation typically yields limited functional advantages in older adults who already ingest sufficient quantities of dietary protein. When administered in conjunction with exercise, whey protein supplementation may offer context-specific support, particularly for individuals exhibiting low habitual protein intake, anabolic resistance, frailty, or impaired metabolic health. Nevertheless, the additive effects of whey protein are heterogeneous and are significantly modulated by variables such as protein dosage and quality, exercise intensity, baseline nutritional status, and adherence to the intervention. Enhancements in molecular and biochemical biomarkers, encompassing anabolic signaling, oxidative stress, inflammation, cellular hydration, and microRNA expression, do not always translate into superior functional or cognitive outcomes, thereby underscoring the complexity inherent in biological adaptation during the aging process. Significantly, the muscle–brain–metabolism axis provides a useful conceptual framework for elucidating the manner in which exercise-induced muscular adaptations and specific nutritional interventions may synergistically impact systemic metabolism and cognitive function. However, this framework is still evolving and requires further empirical validation. Exploiting this axis may facilitate the formulation of more precise, individualized strategies designed to sustain physical autonomy, metabolic robustness, and cognitive well-being throughout the aging process. Collectively, these findings support a model in which exercise constitutes the cornerstone of healthy aging strategies, with whey protein supplementation acting as a supplementary, tailored approach rather than a one-size-fits-all remedy. Future studies focused on precision and mechanistic human research are needed to better define integrated nutrition–exercise interventions and to translate emerging biological knowledge into efficacious clinical and public health guidelines.
